# Suppression of Tau Phosphorylation Induces Neurotoxicity, Causing Developmental Defects and Degeneration in *C. elegans*

**DOI:** 10.3390/cells15090793

**Published:** 2026-04-27

**Authors:** Man Pok Lu, Yi Rong, Jingyi Wang, Xiaochun Yu, Hongjiang Liu, Yingjie Wu, Minxing Zhang, Yining Chen, Yidong Li, Yuner Yan, Aiden Liu, Zhaoyu Li

**Affiliations:** 1 Queensland Brain Institute, The University of Queensland, Brisbane 4072, Australia; 2 Department of Neuroscience, City University of Hong Kong, Hong Kong SAR, China; 3 Institute of Fluid Physics, China Academy of Engineering Physics, Mianyang 621999, China; 4 Melbourne Brain Centre Imaging Unit, Department of Radiology, The University of Melbourne, Melbourne 3010, Australia; 5 College of Engineering, University of Michigan, Ann Arbor, MI 48109, USA

**Keywords:** *C. elegans*, tau phosphorylation, hTauE14, hTauAP, C-terminal region (CTR), microtubule-binding domain (MTB)

## Abstract

**Highlights:**

**What are the main findings?**

- Tau hypophosphorylation (hTauAP) induces severe neurotoxicity in *C. elegans*, leading to aberrant neurites and behavioural deficits.
- hTauAP-induced toxicity requires both the microtubule-binding domain (MTB) and the C-terminal region (CTR).

**What are the implications of the main findings?**

- Broadly suppressing tau phosphorylation as a therapeutic strategy is risky, as it may induce neurite abnormalities and neurotoxicity.

**Abstract:**

Tau hyperphosphorylation is a hallmark of tauopathies and is closely associated with neurodegeneration. While targeting kinases and phosphatases to suppress tau phosphorylation has become an increasingly attractive therapeutic approach, the functional significance of tau phosphorylation and the potential risks of suppressing this process are not fully understood. Using *C. elegans*, we introduced non-phosphorylatable tau mutations (hTauAP) to model the suppression of tau phosphorylation. Unexpectedly, we found that hTauAP induced severe neurotoxicity, resulting in behavioural deficits and severe neurite abnormalities. This neurotoxicity is associated with excessive accumulation of hTauAP on microtubules, leading to both neurite developmental defects and adult neurite degeneration. The neurotoxic effects of hTauAP require its microtubule-binding domain (MTB) and are primarily driven by the loss of phosphorylation in the C-terminal region (CTR). Removing either domain reduces microtubule association and suppresses toxicity. Within CTR, suppressing phosphorylation at S396 or S404 is critical for neurotoxicity. These findings highlight the essential role of tau phosphorylation in neuronal function and underscore the potential risks of broadly suppressing tau phosphorylation as a therapeutic strategy.

## 1. Introduction

Tauopathies are a heterogeneous group of neurodegenerative disorders characterized by the accumulation of tau-positive aggregates, including Alzheimer’s disease (AD), frontotemporal dementia (FTD) and Pick’s disease [1,2,3,4]. Collectively, these disorders affect over 50 million individuals worldwide, yet effective treatments remain elusive [5]. Tau hyperphosphorylation is widely recognized as a key driver of aggregate formation and disease progression [3,4,6,7]. In AD, the presence of neurofibrillary tangles strongly correlates with cognitive decline and neuronal loss, with these pathological structures consistently composed of hyperphosphorylated tau [1,2,3,4,8].

Tau hyperphosphorylation occurs at multiple sites, with distinct phosphorylation patterns leading to different functional outcomes. Tau phosphorylation also occurs under physiological conditions. Under physiological conditions, robust tau phosphorylation at the AT8, pT217, and pS422 epitopes defines a characteristic mitotic phospho-Tau signature during mitosis [9]. Under pathological conditions, modifications at residues such as S202, T205, T212, S214, T217, S262, and S422 promote tau aggregation and lead to the accumulation of SDS-insoluble tau species, whereas phosphorylation at S214, T241, S396, and S404 reduces tau’s affinity for microtubules [10]. In *Drosophila*, pseudo-hyperphosphorylation at 14 SP/TP kinase target sites (T111, T153, T175, T181, S199, S202, T205, T212, T217, T231, S235, S396, S404, and S422), commonly termed “disease-associated” sites, induces severe cytotoxicity, leading to disrupted ommatidial arrangement and a characteristic rough eye phenotype, despite tau remaining soluble [11,12]. In *C. elegans*, pseudo-hyperphosphorylation at multiple sites (S198, S199, S202, T231, S235, S396, S404, S409, S413) promotes neuronal toxicity and aging-related neurodegeneration without detectable tau aggregation [13]. Given the central role of tau hyperphosphorylation in disease pathology, targeting tau phosphorylation has become an increasingly attractive therapeutic strategy for AD [14,15]. Key regulatory molecules, including glycogen synthase kinase 3 beta (GSK-3β), cyclin-dependent kinase 5 (CDK5), tau-tubulin kinases (TTBK), microtubule affinity-regulated kinases (MARK), and protein phosphatase 2A (PP2A), have been identified as potential therapeutic targets [14,15].

However, the role of tau phosphorylation in neuronal toxicity remains controversial. While hyperphosphorylated tau is linked to aggregation and toxicity, emerging evidence suggests that suppressing tau phosphorylation (hypophosphorylation) may also be harmful. In *Drosophila*, hypophosphorylated tau disrupts axonal transport and neurohormone release, leading to significant neurotoxicity [16]. Moreover, age-dependent dopamine neuron loss has been specifically associated with hypophosphorylated but not hyperphosphorylated tau [17]. These findings raise important concerns about the potential risks of therapeutic strategies aimed at reducing tau phosphorylation.

To directly investigate the effects of tau hypophosphorylation on neuronal function, we utilized *C. elegans* as a model system to express non-phosphorylatable tau in neurons. Our results revealed that tau hypophosphorylation leads to severe behavioural impairment, primarily due to neurite loss. Notably, this neurotoxicity not only causes neurite developmental defects but also neurite neurodegeneration. Further analysis identifies the microtubule-binding domain (MTB) and phosphorylation sites such as S396 and S404 within the C-terminal region (CTR) as critical regulators of this process. Our study underscores the potential risks of targeting tau phosphorylation as a therapeutic approach and highlights the necessity of maintaining a balanced phosphorylation state for proper neuronal function.

## 2. Materials and Methods

*C. elegans Strains and Maintenance: C. elegans* strains were maintained at 20 °C on Nematode Growth Medium (NGM) agar plates seeded with *E. coli* OP50, as previously described [18]. Conditional expression of hTauAP was achieved as previously described [19]. In brief, worms were exposed to NGM plates containing 0.1 ng/μL doxycycline hyclate starting at different stages (egg stage or L4 stage). Day 2 and Day 4 adult worms, with or without doxycycline treatment, were then mounted for imaging.

Transgenic strains harbouring extrachromosomal arrays were generated via microinjection. Injections were performed with an inverted Olympus CX51 microscope equipped with an Eppendorf FemtoJet 4i (Eppendorf, Hamburg, Germany). Plasmid concentrations between 1 and 25 ng/μL were used. Strains exhibiting high rates of extrachromosomal array transmission were selected for further testing. Some arrays were integrated into the *C. elegans* genome through the TMP-UV method: 70 worms with extrachromosomal arrays were treated with 30 μg/mL trimethylpsoralen (TMP) for 15 min, then exposed to UVC using a UV crosslinker. About 200 F2 progeny were individually isolated and screened to confirm successful integration. Positive strains were backcrossed five times with N2 wild-type worms to eliminate background mutations. All strains used are detailed in Supplementary Table S1 strain list.

*Plasmid Construction and Transgenic Strains:* hTauWT, hTauE14, and hTauAP were obtained from Addgene [20]. They were amplified and cloned into the *C. elegans* expression vector pBS77 using BglII and NotI. The 1.8 kb *Punc-25* promoter, which drives expression in GABAergic neurons, was amplified from *C. elegans* genomic DNA and inserted into pBS77 using SphI and BamHI. Truncated variants, including hTauAP(ΔNTP), hTauAP(ΔPRR), hTauAP(ΔMTB), and hTauAP(ΔCTR), were synthesized by IDT (Integrated DNA Technologies) and cloned into pBS77 via BglII and NotI. EMTB was amplified from EMTB-3XGFP [21] and inserted into the *pBS77::Punc-25::mKate2* plasmid. Conditional expression of hTauAP was achieved using the Tet/Q Hybrid System [19] by inserting hTauAP into the *TRE-Δpes-10-GFP* plasmid (Addgene, Watertown, MA, USA).

*Optogenetics and Behavioural Assays:* Optogenetic stimulation and behavioural analysis were conducted with the WormLab system (MBF Bioscience, Williston, VT, USA). The system’s built-in red light was used to activate Chrimson in the ASH neuron. Because *C. elegans* cannot produce all-trans-retinal (ATR) naturally, we supplemented the plates with exogenous ATR to ensure proper light sensitivity. In brief, six to eight L4-stage worms were transferred to NGM plates with 5 μM ATR and allowed to grow for one generation. Subsequently, L4 worms were selected and transferred to fresh ATR plates containing OP50, and they were allowed to grow to D1 adults. These worms were subsequently tested on standard NGM plates without OP50 and ATR. For each behavioural test, ~10 worms were placed on assay plates. Worms were gently tapped to synchronize forward movement immediately before recording, and 30 s videos were captured at a rate of 7.5 Hz using the built-in camera of Wormlab. A 590 nm red light stimulus was presented for two seconds at the 10 s point. The experiments were repeated at least three times. Behavioural metrics, including locomotory speed and body contraction, were measured and analysed with the Wormlab system.

*Confocal Imaging and Data Analysis:* Confocal imaging was conducted at the Queensland Brain Institute’s Advanced Microscopy Facility using a spinning disk confocal microscope (Diskovery; Andor Technology, Belfast, UK) built around a Nikon Ti-E body (Nikon Corporation, Tokyo, Japan). Imaging was performed with a CFI Apo Lambda S LWD 40×WI/1.15 NA water-immersion objective (Nikon Corporation, Tokyo, Japan), and a Plan Apo Lambda 100×/1.45 NA oil-immersion objective (Nikon Corporation, Tokyo, Japan). The system was equipped with a Zyla 4.2 sCMOS camera (Andor Technology, Belfast, UK) and controlled by Nikon NIS software (Version 5.21.03) (Nikon Corporation, Tokyo, Japan).

To image D-type GABAergic motor neurons, worms were immobilized with 5 mM tetramisole hydrochloride (Sigma-Aldrich, St. Louis, MO, USA) and mounted on 6% agarose pads. The neurites of these GABAergic motor neurons are on the right side of the worm body. To improve imaging quality, we positioned worms laterally with their right sides facing the objective. 3D imaging was performed in segments from the tail to the head of the worm body, which were then projected using maximum intensity and stitched together in ImageJ (Version 1.54n 17 February 2025) to obtain the full GABAergic motor neuron pattern. Protein steady-state levels were quantified using whole-worm fluorescence intensity. Briefly, stitched whole-worm images were thresholded to identify neuronal cell bodies and neurites. To minimize autofluorescence contamination, the threshold was set above the majority of the autofluorescence signal. Residual autofluorescent regions were manually excluded, and regions of interest (ROIs) were defined for quantification of fluorescence intensity (Figure S2A). Fluorescence intensity within ROIs was measured using the Analyze Particles function in ImageJ. Whole-worm fluorescence intensity was calculated as the sum of fluorescence intensity within all ROIs divided by the total ROI area. For all imaging at different stages, the same imaging conditions, including exposure time and laser power, were used to allow consistent comparisons across different conditions.

Neurite morphology was categorized as described: Normal neurites extend from the ventral to dorsal side without looping or beading. Short neurites extend from the ventral side but fail to reach the dorsal side. Looping neurites display circular structures along the length of the neurite. Beading neurites exhibit a series of small bead-like structures along the neurite.

To assess neurite growth, 20 Day 1 adult worms were transferred to OP50-seeded plates and allowed to lay eggs for 2 h before being removed. After 24 h, larval worms were mounted on slides and imaged using a 100× objective. Vaseline was applied to the edges of the cover glass to prevent evaporation. The same worms were reimaged six hours later to track neurite extension. Growth cones were classified as normal if they exhibited a tree-like or mushroom-shaped morphology.

Nocodazole was used to inhibit microtubule function in *C. elegans* neurons [22]. Day 1, adult worms were transferred to NGM plates containing 5 µM nocodazole and allowed to lay eggs for 3 h, after which the adults were removed. Worms were raised on plates with nocodazole and were imaged at the Day 1 adult stage. Control worms were raised on plates without nocodazole.

## 3. Results

### 3.1. Hypophosphorylation of Tau Causes Neurotoxicity That Impairs Behaviour in C. Elegans

Phosphorylation at Serine-Proline/Threonine-Proline (SP/TP) sites in human tau is implicated in tauopathy-associated neurodegeneration [11]. To investigate the role of SP/TP phosphorylation in tau-induced neuronal toxicity, we expressed human tau 0N4R (hTauWT) in *C. elegans*, introducing mutations at all 14 SP/TP sites (T111, T153, T175, T181, S199, S202, T205, T212, T217, T231, S235, S396, S404, and S422), commonly termed “disease-associated” sites, to either glutamate (hTauE14) or alanine (hTauAP) to mimic hyperphosphorylation and hypophosphorylation, respectively [11,12]. In Alzheimer’s disease (AD), phosphorylated tau accumulates prominently in GABAergic neurons of the dentate gyrus (DG) [23]. To model tau phosphorylation states in *C. elegans*, we expressed hTauE14 and hTauAP specifically in GABAergic neurons. These neurons in the ventral nerve cord exhibit a stereotyped morphology, with neurites extending from the ventral to the dorsal side, and play a crucial role in regulating muscle relaxation during spontaneous movement and avoidance behaviour [24].

**Figure 1:**
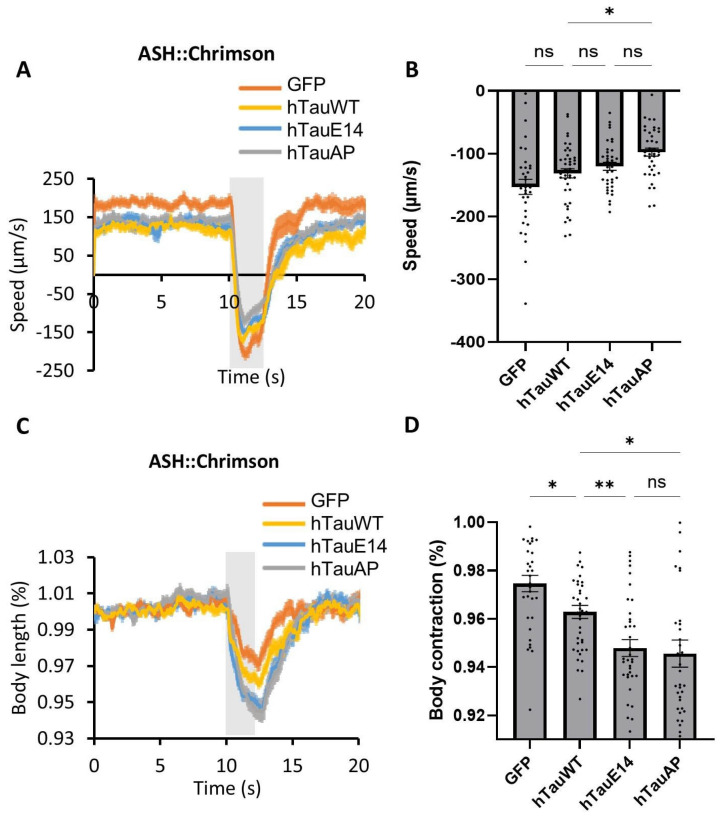
Expression of hTauAP and hTauE14 in *C. elegans* GABAergic neurons leads to behavioural dysfunction. (**A**). Effects of hTauAP and hTauE14 expression on ASH::Chrimson-triggered escape behaviour. A two-second red light pulse (gray bar, 590 nm) was applied to activate the nociceptive ASH neuron expressing Chrimson. (**B**). Quantifications of reversal speed (negative velocity) in (**A**). Data are presented as mean ± SEM, *n* = 33, 40, 37, 41 from left to right. * *p* = 0.014 (hTauWT vs. hTauAP), *p* = 0.243 (GFP vs. hTauWT), 0.748 (hTauWT vs. hTauE14), 0.195 (hTauE14 vs. hTauAP), one-way ANOVA with Tukey’s post hoc test. (**C**). Effects of hTauAP and hTauE14 expression on ASH::Chrimson-triggered body shrinkage. The gray bar indicates red light (2 s, 590 nm) activation. (**D**). Quantifications of body contraction in (**C**). Body length before contraction was normalized to 1. Data are presented as mean ± SEM, *n* = 33, 40, 37, 41 from left to right. * *p* = 0.05 (GFP vs. hTauWT), 0.044 (hTauWT vs. hTauAP), ** *p* = 0.006 (hTauWT vs. hTauE14), *p* > 0.999 (hTauE14 vs. hTauAP, ns: not significant), one-way ANOVA with Tukey’s post hoc test.

To investigate the functional consequences of tau phosphorylation in GABAergic neurons, we expressed hTauWT, hTauE14, and hTauAP and quantified animal behaviour following optogenetic activation of the nociceptive neurons in *C. elegans*. We selectively stimulated nociceptive neuron ASH using Chrimson, a red-light-activated channelrhodopsin [25]. In wild-type worms, the activation of ASH typically elicited a rapid backward escape response (Figure 1A). In contrast, worms expressing hTauE14 exhibited a significant reduction in escape speed along with excessive body contraction (Figure 1A–D), indicating impaired muscle relaxation. These defects suggest dysfunctions in GABAergic neurons, which are essential for muscle relaxation and coordinated motor responses. Unexpectedly, worms expressing hTauAP also displayed severe defects in these behaviours (Figure 1A–D), suggesting that tau hypophosphorylation also leads to neuronal dysfunction. Notably, this toxicity was not restricted to GABAergic neurons, as panneuronal expression of either hTauE14 or hTauAP resulted in reduced body size during development (Figure S1). These findings demonstrate that not only hyperphosphorylation but also hypophosphorylation of tau causes neuronal toxicity.

### 3.2. Hypophosphorylation of Tau Leads to Aberrant Neurites and Excessive Accumulation of Tau on Microtubules

**Figure 2:**
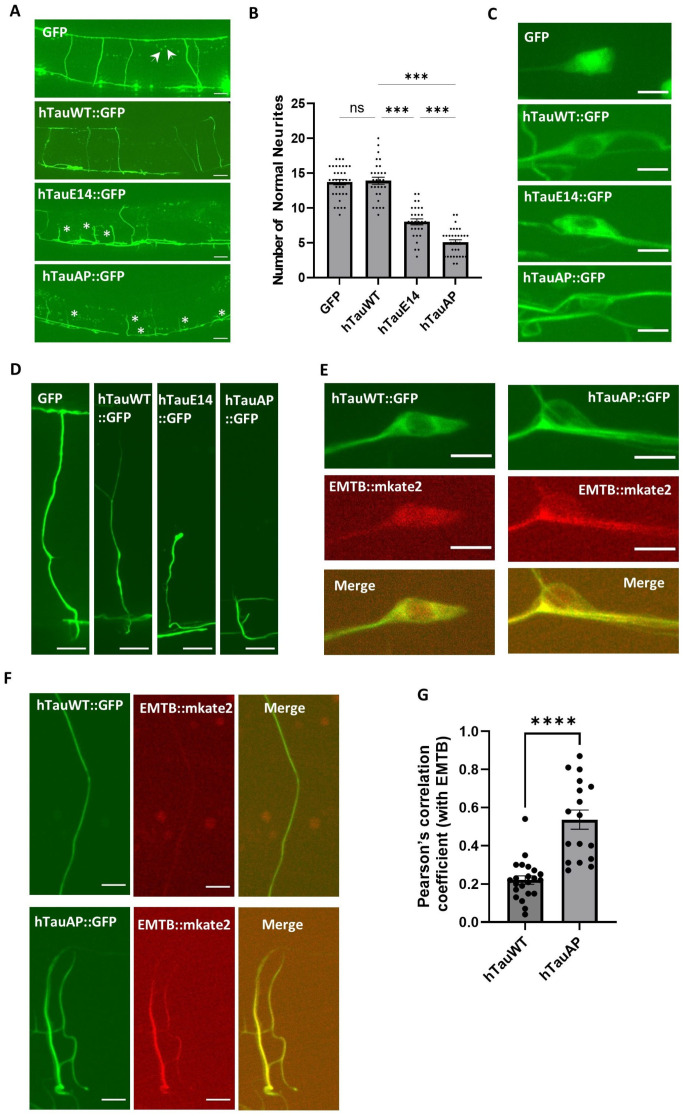
hTauAP causes short neurites and promotes its accumulation along microtubules. (**A**) Effects of hTauAP and hTauE14 expression on neurite morphology. Normal neurites extend from the ventral to the dorsal side. Asterisks indicate aberrant neurites that fail to reach the dorsal side. Arrows indicate autofluorescence unrelated to neurons. Scale bar: 20 µm. (**B**) Quantifications of worms exhibiting normal neurite projections under different tau phosphorylation states. Data are presented as mean ± SEM, *n* = 35,29,29,33 from left to right. *p* = 0.99 (GFP vs. hTauWT, ns: not significant), *** *p* < 0.001 (hTauWT vs. hTauAP, hTauWT vs. hTauE14, hTauE14 vs. hTauAP), one-way ANOVA with Tukey’s HSD test. (**C**) Fluorescence distribution of hTauAP and hTauE14 in neuronal somas. Note that hTauAP worms showed bright fibre-like patterns. Scale bar: 4 µm. (**D**) Neurite morphology in worms expressing hTauAP and hTauE14. Scale bar: 10 µm. (**E**) hTauAP accumulates along microtubules in the soma. EMTB::mKate2 as a marker for microtubules. Scale bar: 4 µm. (**F**) hTauAP accumulates along microtubules in neurites, visualized using EMTB labelling. Scale bar: 10 µm. (**G**) Pearson’s correlation coefficient (R) from EMTB colocalization. Data are presented as mean ± SEM, *n* = 22, 17. **** *p* < 0.0001, unpaired T-test.

As the neuronal toxicity associated with tau hypophosphorylation remains poorly understood, this study mainly focuses on this topic and seeks to elucidate the underlying mechanisms. To investigate hypophosphorylation-induced toxicity, we examined GABAergic neurons using confocal microscopy. In *C. elegans*, the cell bodies of GABAergic motor neurons are positioned along the ventral side, projecting neurites dorsally via commissures [24]. In animals expressing either GFP alone or wild-type human tau, neurites exhibited normal extension toward the dorsal side (Figure 2A,B,D). In contrast, worms expressing hTauAP showed abnormal neurite morphology, with notably shorter neurites that could not extend dorsally, and exhibited more severe defects than those in hTauE14 (Figure 2A,B,D), indicating significant neuronal toxicity. To test whether differences in expression levels contribute to the observed neurite abnormalities, we injected plasmids encoding GFP, hTauWT, hTauE14, and hTauAP at equal concentrations. Under these conditions, GFP and hTauWT consistently exhibited higher fluorescence intensities than hTauE14 and hTauAP. Comparable fluorescence levels were only achieved by reducing the injection concentration of hTauWT (Figure S2A–D). These results indicate that the neurite defects observed in hTauE14 and hTauAP are not due to differences in expression levels, and suggest that protein toxicity may influence steady-state protein levels.

Proper neurite structures are essential for these GABAergic neurons to receive synaptic inputs and generate inhibitory outputs to innervate dorsal body muscles [24]. The loss of neurites is likely to disrupt both the inputs to these GABAergic neurons and the inhibitory outputs to muscles, thereby impairing muscle relaxation and contributing to defective escape behaviour and excessive body contraction (Figure 1A–D).

Additionally, at the cellular level, non-phosphorylatable tau (hTauAP) displayed a distinct distribution pattern in both the soma and neurites compared to hTauE14- and hTauWT. It formed highly accumulated, elongated, fiber-like structures with bright fluorescence (Figure 2C). These structures occasionally exhibited rigid, branching segments with discontinuities along the neurites and within neuronal cell bodies, in contrast to the more soluble state of hTauWT (Figure 2B,C). To investigate whether these structures in hTauAP were associated with microtubules, we labelled microtubules with EMTB, a microtubule-binding domain from ensconsin [21]. The EMTB signal was significantly enhanced and colocalized with the bright fibre-like structures formed by hTauAP in both neuron soma and neurites (Figure 2E–G), indicating that the accumulated hTauAP is associated with microtubules. This is consistent with previous studies that hTauAP has a significantly higher binding affinity for microtubules [16]. These findings suggest that tau hypophosphorylation disrupts neurite structure, alters microtubule binding patterns, and induces severe neuronal toxicity in *C. elegans*.

### 3.3. hTauAP-Induced Neuronal Toxicity Results in Both Neurite Developmental Defects in Larvae and Neurite Degeneration in Adults

**Figure 3:**
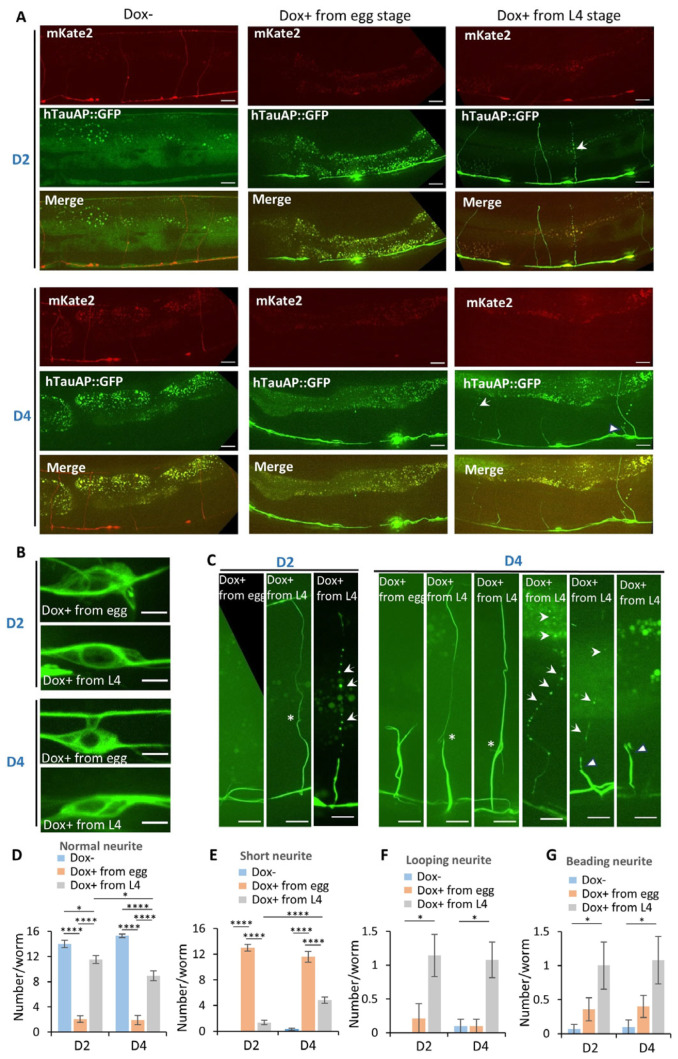
hTauAP-induced neurotoxicity causes both developmental defects and degeneration to neurites. (**A**) Conditional induction of hTauAP expression from different developmental stages (egg stage or the larval L4 stage) via doxycycline (Dox) resulted in varying degrees of neurite defects. Images were captured at days 2 (D2) and day 4 (D4) of adulthood. Arrows indicate beading neurites. Scale bar: 20 µm. (**B**) hTauAP accumulation along microtubules in the soma remains unaffected by conditional induction at different developmental stages. Scale bar: 4 µm. (**C**) Developmental-stage-dependent hTauAP expression led to different neurite defects. Asterisks indicate looping neurites, arrows mark beading neurites, and triangles denote shortened neurites. The brightness of images was adjusted to better visualize the beading neurites. Scale bar: 10 µm. (**D**–**G**) Quantification of normal neurites (**D**), shortened neurites (**E**), looping neurites (**F**), and beading neurites (**G**) in worms with conditional hTauAP expression at different developmental stages. *n* = 12, 14, 14, 10, 10, 13 from left to right in (**D**–**G**) * *p* = 0.013 (D2, Dox- vs. Dox + from L4), 0.034 (Dox+ from L4, D2 vs. D4), **** *p* < 0.0001 in (**D**); **** *p* < 0.0001 in (**E**); * *p* = 0.018 (D2, Dox- vs. Dox+ from L4), 0.030 (D4, Dox- vs. Dox+ from L4) in (**F**); * *p* = 0.046 (D2, Dox- vs. Dox+ from L4), 0.029 (D4, Dox- vs. Dox+ from L4) in (**G**), two-way ANOVA test. Data are presented as mean ± SEM.

We then investigated whether hTauAP-induced neuronal toxicity results from neurodegeneration or developmental defects. To distinguish between these possibilities, we utilized a conditional expression system controlled by the chemical doxycycline (Dox) [19], allowing us to activate hTauAP expression either before or after D-type motor neuron development. *C. elegans* progresses through four larval stages (L1–L4) before reaching adulthood, and D-type motor neuron neurite development is complete by the L2 stage [26,27]. In the absence of Dox, hTauAP GFP fluorescence was low, and soluble mKate fluorescence indicated normal neurite morphology (Figure 3A). Upon Dox treatment starting from different stages, either from the egg stage or the larval L4 stage, we observed robust hTauAP GFP expression, confirming successful induction (Figure 3A).

To distinguish whether the observed neurite shortening results from neurodegeneration or developmental defects, we induced hTauAP expression either before neurite development (from the egg stage) or after its completion (from L4) and performed imaging at day 2 (D2) and day 4 (D4) of adulthood. If neurodegeneration were the primary cause, neurite defects should appear at D2 and worsen by D4 in worms induced from L4, whereas those induced from either the egg or L4 stage should exhibit no significant differences at each time point. Conversely, if the defects stemmed from developmental abnormalities, induction from the egg stage would lead to more severe neurite defects compared to L4 induction, and worms induced from L4 should exhibit no defects at D2 or D4.

Our findings revealed that neurite loss was more pronounced in worms induced from the egg stage than from L4, suggesting a developmental impairment (Figure 3A-middle panels, 3D). Degeneration also contributed to this phenotype. In worms induced from L4, neurite loss was significant at D2 and became more severe by D4 (Figure 3A-right panels, 3D). These results suggest that both developmental defects and degeneration contribute to neurite aberration in these neurons.

A closer examination of neurite morphology provided further insights into hTauAP toxicity. Despite comparable hTauAP accumulation with microtubule-associated patterns in the soma of both groups (Figure 3B), the effects on neurites differed. In worms induced from the egg stage, neurites were very short, exhibiting bright fibre-like structures along their length at both D2 and D4 (Figure 3C,E). In contrast, worms induced from L4 displayed distinct phenotypes. In this group, although the majority of neurites extended to the dorsal side, some exhibited a looping phenotype, forming circular structures, while others showed a beading phenotype, characterized by a series of small beads along the neurite, a hallmark of neurite degeneration (Figure 3C,F–G). These defects became more pronounced with age, with some beading neurites eventually undergoing degeneration and shortening (Figure 3C,E). Together, these results indicate that hTauAP-induced toxicity not only disrupts neurite development during larval stages but also leads to progressive neurite degeneration in adulthood.

We then examined how it caused developmental defects. Growth cones are dynamic structures that direct neurite extension in response to attractive and repulsive cues, a process dependent on precise microtubule dynamics. Disruptions in microtubule function can impair growth cone structure and hinder neurite extension [28,29]. To assess this, we analysed the growth cones of VD neurons at the L2 stage, as these GABAergic neurons are born postembryonically, allowing real-time imaging. In hTauAP-expressing worms, growth cones appeared severely shrunken and exhibited defective morphology, with minimal extension toward the dorsal side (Figure S3A,B). Additionally, neurite growth and extension were significantly impaired compared to hTauWT-expressing worms (Figure S3A,C). Given hTauAP’s much higher microtubule-binding affinity and its excessive accumulation around microtubules, these defects in neurite growth are likely associated with excessive hTauAP binding caused microtubule dysfunction. Indeed, pharmacological inhibition of microtubule function in hTauWT worms using nocodazole induced aberrant neurite morphology similar to that observed in hTauAP worms (Figure S3D–E).

### 3.4. hTauAP-Induced Neural Toxicity Requires the Microtubule-Binding Domain (MTB) and the C-Terminal Region (CTR)

**Figure 4:**
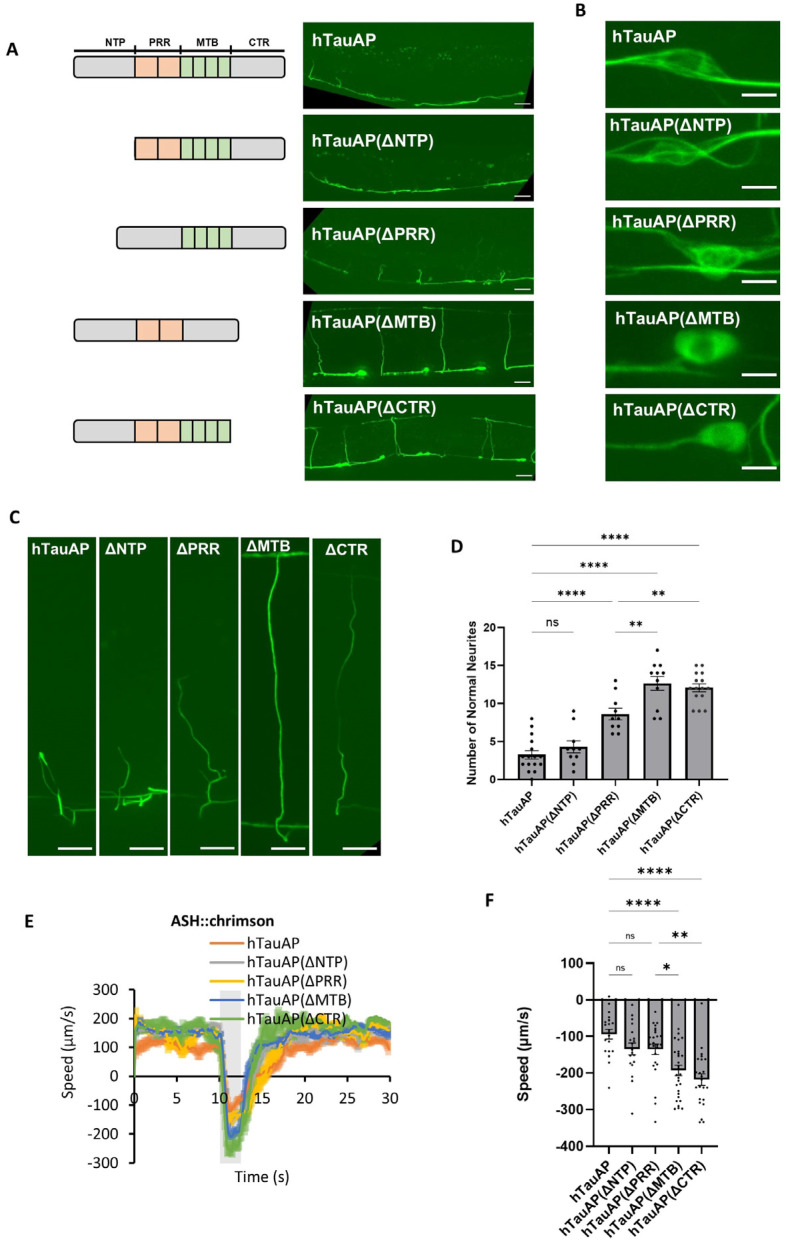
Domain-specific contributions to hTauAP-induced neurotoxicity. (**A**) Deletion of the microtubule-binding domain (MTB) or C-terminal region (CTR) suppressed hTauAP-induced neurite aberrant phenotypes. All truncated hTau variants were fused to GFP. Scale bar: 20 µm. (**B**) Effects of domain deletions on hTauAP accumulation along microtubules in the soma. Scale bar: 4 µm. (**C**) Neurite morphology in worms expressing hTauAP with different domain deletions. Scale bar: 10 µm. (**D**) Quantification of worms with normal neurite projections across different domain deletions. *n* = 16, 10, 10, 11, 15. *p* = 0.817 (hTauAP vs. hTauAP(ΔNTP), ns: not significant); ** *p* = 0.003 (hTauAP(ΔPRR) vs. hTauAP(ΔMTB)), 0.008 (hTauAP(ΔPRR) vs. hTauAP(ΔCTR)); **** *p* < 0.0001, one-way ANOVA with Tukey’s HSD test. (**E**). Optogenetic activation of ASH neurons-induced escape behavior in worms expressing hTauAP with different domain deletions. The gray bar indicates the red light activation period. (**F**) Quantification of reversal speed in (**E**). Data are presented as mean ± SEM, * *p* < 0.05, ** *p* < 0.01, **** *p* < 0.0001 (one-way ANOVA with Tukey’s HSD test), *n* = 20, 19, 25, 29, 23 from left to right, *p* = 0.391 (hTauAP vs. hTauAP(ΔNTP)), 0.315 (hTauAP vs. hTauAP(ΔPRR)); * *p* = 0.030 (hTauAP(ΔPRR) vs. hTauAP(ΔMTB)); ** *p* = 0.001 (hTauAP(ΔPRR) vs. hTauAP(ΔCTR)); **** *p* < 0.0001, one-way ANOVA with Tukey’s HSD test.

The hTau0N4R protein consists of four functional domains: the N-terminal Projection (NTP), the proline-rich region (PRR), the microtubule-binding domain (MTB), and the C-terminal region (CTR) (Figure 4A, left panel) [30]. Among its 14 phosphorylatable SP/TP sites, 10 are located in the PRR (T153, T175, T181, S199, S202, T205, T212, T217, T231, S235), one in the NTP (T111), and three in the CTR (S396, S404, S422), while none are found in the MTB [12].

To identify the domain responsible for hTauAP-induced neuronal toxicity, we generated truncated versions of hTauAP by selectively removing individual domains (Figure 4A, left panel). Given that the proline-rich region (PRR) contains over 70% of the phosphorylation sites, we initially investigated its contribution by deleting this domain. Surprisingly, despite encompassing the majority of phosphorylation sites, PRR deletion only mildly alleviated neurite defects (Figure 4A,D) and had little effect on the formation of fibre-like aggregates in the soma and short neurites (Figure 4B–C). Similarly, deletion of the N-terminal region (NTP) failed to suppress hTauAP-induced neurite defects as well as the aggregation patterns in soma and neurites (Figure 4A–D).

In contrast, removal of the C-terminal region (CTR) significantly rescued neurite defects, resulting in a greater number of neurites extending toward the dorsal side (Figure 4A,C,D). Moreover, CTR deletion resulted in a more soluble hTauAP state (Figure 4B), suggesting that this region plays a crucial role in driving toxicity. Unexpectedly, despite lacking serine-proline/threonine-proline (SP/TP) phosphorylation sites, deletion of the microtubule-binding (MTB) domain strongly suppressed neurite defects and led to a soluble state in the soma, similar to the effects of CTR deletion (Figure 4A–D). The rescue effects were not due to differences in protein steady-state levels, as the less toxic variants hTauAP(ΔMTB) and hTauAP(ΔCTR) were expressed at higher levels in neurons (Figure S4A), similar to hTauWT, which is expressed at higher levels than hTauAP. These findings indicate that microtubule binding is a key determinant of hTauAP-induced neurotoxicity and suggest that this function may be regulated by the CTR. Although the MTB domain itself lacks phosphorylation sites, the phosphorylation of residues within the CTR appears essential for modulating this process.

Our results suggest that suppression of phosphorylation, particularly in the CTR, alters MTB function, leading to excessive binding of non-phosphorylated tau to microtubules. This ultimately results in developmental defects in neurite growth and neurite loss. Behavioural data further support this conclusion, as deletion of both the CTR and MTB rescued locomotion defects in hTauAP-expressing worms, highlighting their essential roles in hTauAP-induced neuronal toxicity (Figure 4E,F). The significance of the MTB and CTR domains was also evident in body size assays, where pan-neuronal hTauAP-expressing worms exhibited reduced body size, a phenotype that was significantly rescued upon deletion of either the MTB or CTR domain (Figure S4B).

### 3.5. Suppression of Phosphorylation at S396 and S404 in the C-Terminal Region (CTR) Is Sufficient to Cause Neural Toxicity

**Figure 5:**
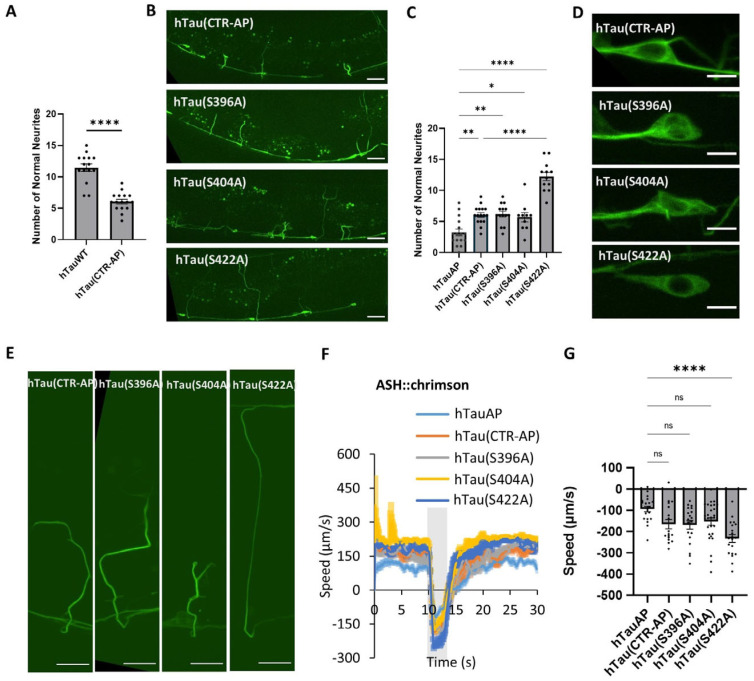
Contribution of different phosphorylation sites in the CTR domain to neuronal toxicity. (**A**) Quantification of normal neurite morphology in worms with hypophosphorylation only in the CTR domain (hTau(CTR-AP)). *n* = 15, 16 from left to right, **** *p* < 0.0001, unpaired T-test. Data are presented as mean ± SEM. (**B**) Effects of suppressing phosphorylation at different sites in the CTR domain on neurite loss. The three phosphorylation sites S396, S404, and S422 were each or all mutated to Alanine to suppress their phosphorylation. Scale bar: 20 µm. (**C**) Quantification of normal neurite morphology in worms expressing hTau with different S396, S404, and S422 sites in CTR domain mutated to Alanine. *n* = 16,16,14,10,12. * *p* = 0.031 (hTauAP vs. hTau(S404A)), ** *p* = 0.001 (hTauAP vs. hTau(CTR-AP)), 0.002 (hTauAP vs. hTau(S396A)), **** *p* < 0.0001, one-way ANOVA with Tukey’s HSD test. Data are presented as mean ± SEM. (**D**) Effects of suppressing phosphorylation at different sites in the CTR domain on hTau accumulation along microtubules in soma. Scale bar: 4 µm. (**E**) Effects of suppressing phosphorylation at different sites in the CTR domain on neurite morphology. Scale bar: 10 µm. (**F**) Effects of suppressing phosphorylation at different sites in the CTR domain on ASH::Chrimson-triggered escape behaviour. Grey bar indicates red light activation segment. (**G**) Quantification of reversal speed in (**E**). Data are presented as mean ± SEM, *n* = 20, 17, 19, 28, 20. *p* = 0.081 (hTauAP vs. hTau(CTR-AP)), 0.053 (hTauAP vs. hTau(S396A)), 0.120 (hTauAP vs. hTau(S404A)), ns: not significant; **** *p* < 0.0001 (hTauAP vs. hTau(S422A)), one-way ANOVA with Tukey’s HSD test.

To further investigate the role of SP/TP phosphorylation sites in the CTR domain, we mutated all three sites in CTR to alanine hTau(CTR-AP) to prevent phosphorylation. Worms carrying these mutations exhibited severe neurite loss (Figure 5A). Given that deletion of the CTR domain suppresses hTauAP-induced neurite defects, these results indicate that preventing phosphorylation in the CTR is both necessary and sufficient to drive hTauAP-induced neuronal toxicity.

To delineate the individual contributions of these phosphorylation sites, we selectively substituted S396, S404, or S422 with alanine to mimic a non-phosphorylated state and assessed their effects on neurite morphology. Alanine substitutions at S396 and S404, but not S422, resulted in neurite loss phenotypes comparable to those observed with the triple-site mutation (Figure 5B,C,E). Additionally, hTau proteins carrying S396A or S404A mutations formed fibre-like excessive microtubule-binding structures in the soma and within shortened neurites (Figure 5D–E), suggesting that suppression of phosphorylation at S396 or S404 but not S422 is responsible for neuronal toxicity. The phenotypes were not due to differences in protein steady-state levels (Figure S5). The results are consistent with previous studies that phosphorylation of S396 and S404 sites reduces tau’s binding affinity for microtubules [31,32].

Behavioural assays further support this conclusion. Worms expressing hTauS396A or hTauS404A in GABAergic neurons exhibited significantly reduced escape speed, mirroring the deficits observed in hTauAP- or hTau (CTR-AP)-expressing worms. In contrast, phosphorylation suppression at S422 did not show similar defects in both neurite numbers and escape speed (Figure 5B–G). These findings identified S396 and S404 as key regulatory sites mediating hTauAP-induced neuronal toxicity.

## 4. Discussion

In this study, we utilized *C. elegans* GABAergic neurons as a model to investigate the neuronal toxicity associated with different tau phosphorylation states. We found that hypophosphorylation of tau, achieved by mutating 14 disease-associated SP/TP kinase target sites, resulted in behavioural deficits. This phenotype primarily arises from aberrant neurites resulting from both impaired developmental growth and adult neurite degeneration. The neuronal toxicity is likely associated with excessive in hTauAP-microtubule binding within these neurons. Our findings further suggest that multiple domains within hTau contribute to the neuronal toxicity of hTauAP. In particular, the suppression of phosphorylation at specific sites within the C-terminal region (CTR), such as S396 and S404, appears to alter function in the microtubule-binding domain (MTB), leading to accumulation of hTauAP with microtubules and neuronal dysfunction. These results highlight that hypophosphorylation of tau also induces severe neuronal toxicity, raising concerns about therapeutic strategies that solely target tau phosphorylation.

Tau phosphorylation has long been implicated in the formation of neurofibrillary tangles and tauopathy-associated toxicity. Hyperphosphorylation at specific sites, including Thr50, Thr69, and Thr181, has been shown to propagate across multiple epitopes, leading to extensive phosphorylation and cognitive deficits [33]. However, while tau phosphorylation is often associated with toxicity, this is not always the case. For instance, in Alzheimer’s disease (AD) mouse models, phosphorylation of tau at Thr205 has been shown to be protective, preventing the formation of postsynaptic complexes involved in Aβ-induced excitotoxicity [34]. Furthermore, hyperphosphorylation of tau in certain neuronal populations can confer protective effects. In *Drosophila* dopamine neurons, hyperphosphorylation at the same 14 SP/TP-directed sites (hTauE14) results in reduced neuronal death compared to wild-type tau, whereas hypophosphorylation (hTauAP) leads to significantly greater neuronal loss [17]. Under physiological processes such as mitosis, robust phosphorylation has been observed at the AT8, pT217, and pS422 epitopes, which define a characteristic mitotic phospho-Tau signature [9]. These findings suggest that phosphorylation at specific tau sites may serve a functional or neuroprotective role, and blocking phosphorylation at these sites can instead drive neuronal toxicity.

A similar pattern is observed in *Drosophila* motor neurons, where hypophosphorylation of tau leads to severe impairments in vesicle trafficking and neurohormone release [16]. Consistently, our study revealed that hypophosphorylation of the same 14 SP/TP-directed sites in *C. elegans* motor neurons resulted in neurite loss. Collectively, these findings suggest that the toxicity associated with different tau phosphorylation states may be neuron-type specific. While hyperphosphorylated tau is neurotoxic in retinal neurons, hypophosphorylation appears to be more detrimental in dopamine and motor neurons. These observations raise critical concerns regarding current therapeutic approaches that aim to suppress tau phosphorylation as a treatment for tauopathies.

Our findings further indicate that the neuronal toxicity induced by hTauAP is associated with defects in neuronal development as well as neurodegeneration. Inhibition of tau phosphorylation at the CTR enhances tau-microtubule binding, potentially interfering with microtubule dynamics [16]. Proper microtubule function is essential for neuronal growth, axon formation, and synaptic connectivity. During development, hTauAP may disrupt these processes, ultimately leading to the observed aberrant growth cone and neurite loss phenotype in *C. elegans* neurons.

Of particular interest, our results highlight the potential contributions of S396 and S404 to this process. Suppression of phosphorylation at these two sites led to a neurite loss phenotype similar to that observed in hTauAP-expressing neurons. Intriguingly, previous in vitro studies have demonstrated that phosphorylation at S396 and S404 reduces tau’s binding affinity for microtubules [31,32]. These findings provide critical insights into the molecular mechanisms underlying tau hypophosphorylation-induced neuronal toxicity and underscore the importance of precise regulation of tau phosphorylation for maintaining neuronal health.

Several limitations should be acknowledged in this research. First, our mechanistic analyses were mainly performed in GABAergic neurons, which provide single-neuron resolution for morphological assessment; however, this may not fully represent the complexity of pan-neuronal effects. Second, although our data support enhanced association of hTauAP with microtubules, we did not directly measure microtubule dynamics, and thus cannot conclusively define the nature of microtubule dysfunction. Future studies with direct measurements of microtubule dynamics and transport assays will be important to further elucidate the mechanisms underlying tau hypophosphorylation-induced toxicity.

## Data Availability

The original contributions presented in this study are included in the article/Supplementary Material. Further inquiries can be directed to the corresponding authors.

## Supplementary Materials

The following supporting information can be downloaded at: https://www.mdpi.com/article/10.3390/cells15090793/s1, Figure S1. Effects of hTauE14 and hTauAP on worm body size; Figure S2. Neurite defects in hTauAP and hTauE14 are not caused by protein expression levels; Figure S3. hTauAP impairs growth cone structure and neurite extension; Figure S4. Neuronal defects in hTauAP and hTauE14 are not caused by protein expression levels; Figure S5. Quantification of protein expression levels measured by whole-worm fluorescence intensity in strains with different phosphorylation sites mutated in the CTR domain; Supplementary Table S1. Strain list.

## Abbreviations

The following abbreviations are used in this manuscript:

AD	Alzheimer’s disease
SP/TP	Serine-Proline/Threonine-Proline
hTauWT	Wild-type human Tau0N4R
hTauE14	14 SP/TP mutated to Glutamate
hTauAP	14 SP/TP mutated to Alanine
EMTB	Microtubule-binding domain from ensconsin
Dox	Doxycycline
NTP	N-terminal Projection of human Tau0N4R
PRR	Proline-rich region of human Tau0N4R
MTB	Microtubule-binding domain of human Tau0N4R
CTR	C-terminal region of human Tau0N4R
hTau(CTR-AP)	All three SP/TP sites in CTR mutated to Alanine
