# Supplementary material for: Suppression of Tau Phosphorylation Induces Neurotoxicity, Causing Developmental Defects and Degeneration in C. elegans
Source: Cells. 2026 Apr 27;15(9):793. doi: 10.3390/cells15090793 (PMC13163045; doi:10.3390/cells15090793)
Supplement: Supplementary file 1 [file cells-15-00793-s001.zip › cells-4179245-Supplementary figures.pdf]

Supplementary Figures S1-S5.

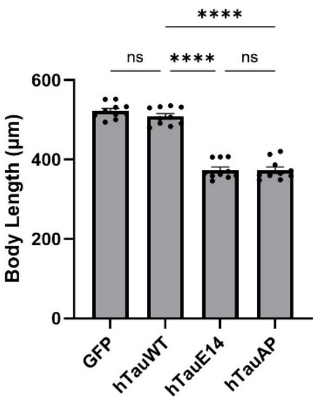

Figure S1. Effects of hTauE14 and hTauAP on worm body size. Worms expressed pan-neuronal hTauWT, E14, and AP under the control of the *Prgef-1* promoter. Body size was measured at 48 hours post-egg. Data are presented as mean ± SEM, $n = 10$ for all the groups. $p = 0.540$ (GFP vs. hTauWT), $p > 0.9999$ (hTauE14 vs. hTauAP), $^{****}p < 0.0001$ (hTauWT vs. hTauE14, and hTauWT vs. hTauAP); ns: not significant; one-way ANOVA with Tukey's HSD test.

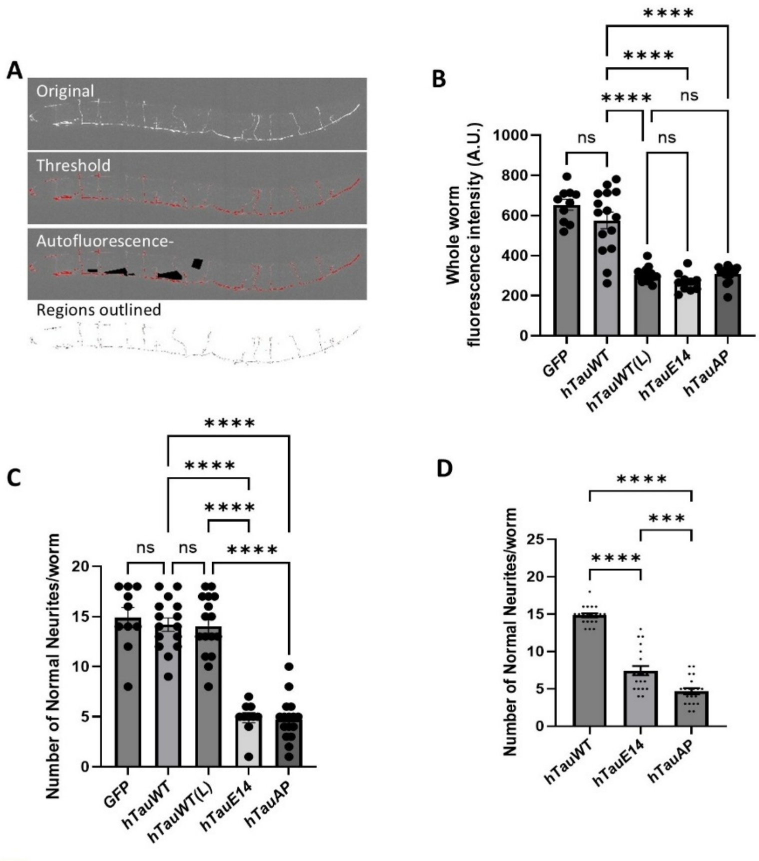

Figure S2. Neurite defects in hTauAP and hTauE14 are not caused by protein expression levels.

(**A**) An example of whole-worm fluorescence measurement is shown. Stitched whole worm images were thresholded (set above the majority of autofluorescence signal) to identify neuronal cell bodies and neurites. Residual autofluorescent regions were manually removed, and regions of interest (ROIs) were then defined for quantification of whole-worm fluorescence intensity.

(**B**) Quantification of protein expression levels measured by whole-worm fluorescence intensity. At the same plasmid injection concentration (50 ng/μl), non- or less-toxic constructs (GFP and hTauWT) consistently exhibited higher fluorescence intensities than toxic constructs (hTauE14 and hTauAP). Lower expression level can also be

achieved in less-toxic strains by reducing the injection concentration (hTauWT(L), 10–20 ng/μl). Data are presented as mean ± SEM (*n* = 10, 15, 16, 10, 16). *p* = 0.2159 (GFP vs hTauWT), 0.8462 (hTauWT(L) vs hTauE14), 0.9997 (hTauWT(L) vs hTauAP), ****$p < 0.0001$; ns: not significant; one-way ANOVA with Tukey's HSD test.

(**C**) Quantification of the number of normal neurites in strains injected at the same concentration or at a reduced concentration (hTauWT(L)). Notably, reducing the injection concentration did not affect the neurite phenotype. Data are presented as mean ± SEM (*n* = 10, 15, 16, 10, 16). P = 0.9644 (GFP vs hTauWT), 0.9999 (hTauWT vs hTauWT(L)), ****$p < 0.0001$; ns: not significant; one-way ANOVA with Tukey's HSD test.

(**D**) Quantification of normal neurite numbers in an independent set of strains (distinct from those in B) revealed similar neurite defects. Data are presented as mean ± SEM (*n* = 20). ****$p < 0.0001$, ***$p = 0.0002$; unpaired two-tailed t-test.

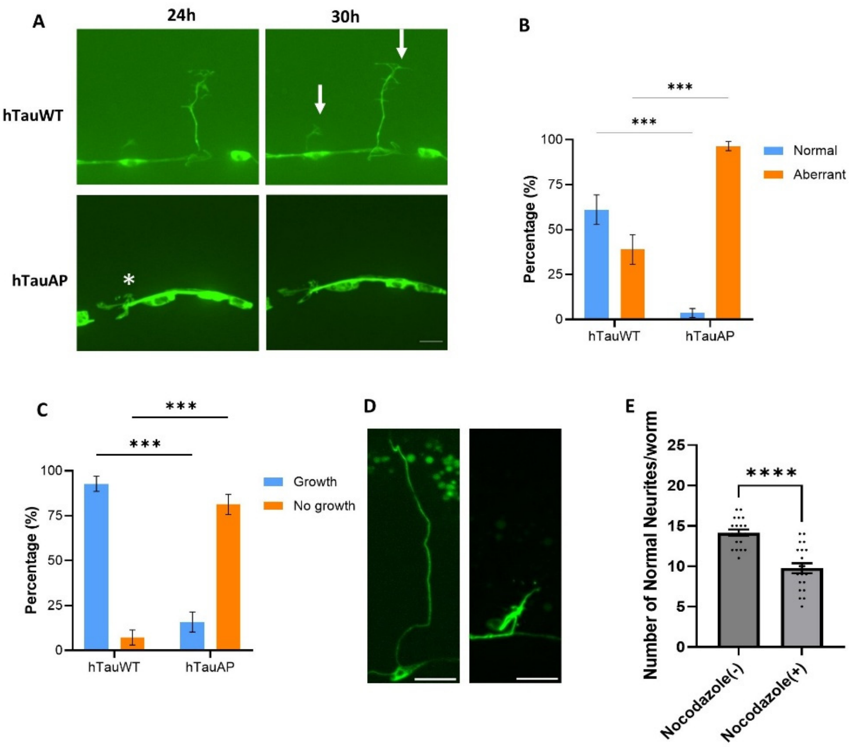

Figure S3. hTauAP impairs growth cone structure and neurite extension.

(**A**) Growth cone morphology in worms expressing hTauWT and hTauAP. By 24 hours post-hatching, worms expressing hTauAP rarely form growth cones. Brightness was adjusted across images to enhance growth cone visibility. White arrows indicate normal extending growth cones, while asterisks mark neurites lacking proper growth cones.

(**B**-**C**) Quantification of normal and aberrant growth cones at 24 hours post-egg (**B**) and neurites that successfully extended between 24 and 30 hours (**C**) in worms expressing hTauWT and hTauAP. *n* = 10 for each group. ***$p < 0.001$, two-way ANOVA test. Data are presented as mean ± SEM.

(**D**) Inhibition of microtubule function by Nocodazole treatments causes neurite defects.

(**E**) Quantification of neurite defects in worms treated with Nocodazole from the egg stage. *n* = 20 for each group. ****$p < 0.0001$, unpaired T-test. Data are presented as mean ± SEM.

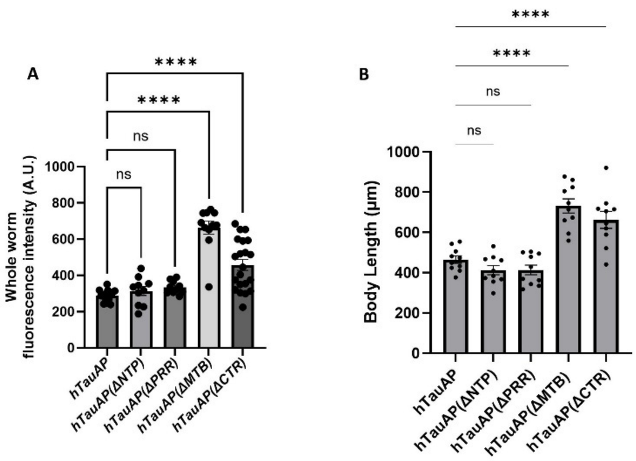

Figure S4. Neuronal defects in hTauAP and hTauE14 are not caused by protein expression levels.**(A)** Quantification of protein expression levels measured by whole-worm fluorescence intensity. At the same plasmid injection concentration (50 ng/μl), non- or less-toxic constructs (hTauAP(ΔMTB) and hTauAP(ΔCTR)) showed higher fluorescence intensities than toxic ones (hTauAP). Data are presented as mean ± SEM (n = 16, 10, 10, 11, 21). p = 0.9917 (hTauAP vs. hTauAP(ΔNTP)), 0.7797 (hTauAP vs. hTauAP(ΔPRR)), **** $p < 0.0001$ (hTauAP vs. hTauAP(ΔMTB), and hTauAP vs. hTauAP(ΔCTR)); ns: not significant; one-way ANOVA with Tukey's HSD test.**(B**) Deletion of the MTB or CTR domain suppresses hTauAP-induced body size defects. Body size was measured at 48 hours post-egg. n = 10 for all the groups. p = 0.534 (hTauAP vs. hTauAP(ΔNTP)), 0.552 (hTauAP vs. hTauAP(ΔPRR)), ****$p < 0.0001$ (hTauAP vs. hTauAP(ΔMTB), and hTauAP vs. hTauAP(ΔCTR)); ns: not significant;one-way ANOVA with Tukey's HSD test. Data are presented as mean ± SEM.

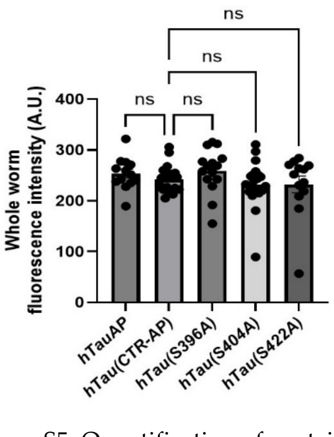

Figure S5. Quantification of protein expression levels measured by whole-worm fluorescence intensity in strains with different phosphorylation sites mutated in the CTR domain. Data are presented as mean ± SEM (n = 13, 20, 15, 22, 13). p = 0.9481 (hTauAP vs hTau(CTR-AP)), 0.7741 (hTau(CTR-AP) vs. hTau(S396A)), 0.9294 (hTau(CTR-AP) vs. hTau(S404A)), 0.9536 (hTau(CTR-AP) vs. hTau(S422A)), ns: not significant; one-way ANOVA with Tukey's HSD test.
