# Supplementary material for: Suppression of Tau Phosphorylation Induces Neurotoxicity, Causing Developmental Defects and Degeneration in C. elegans
Source: Cells. 2026 Apr 27;15(9):793. doi: 10.3390/cells15090793 (PMC13163045; doi:10.3390/cells15090793)
Supplement: Supplementary file 1 [file cells-15-00793-s001.zip › cells-4179245-Supplementary table S1.pdf]

Supplementary table S1: Strain list.

Strains
DNA1 *atgEx1[Punc25::hTauAP(ΔMTB)::GFP]*
DNA2 *atgEx2[Punc25::hTauAP::GFP]*
DNA3 *atgEx3[Punc25::hTauE14::GFP]*
DNA4 *atgEx4[Punc25::hTauWT::GFP]*
DNA5 *atgEx5[Punc25::GFP]*
DNA7 *atgEx7[Punc25::hTauWT::GFP]*
DNA8 *atgEx8[Punc25::hTauE14::GFP]*
DNA9 *atgEx9[Punc25::hTauAP::GFP]*
DNA72 *atgEx72 [Punc25::hTauAP(ΔNTP)::GFP]*
DNA73 *atgEx73[Punc25::hTauAP(ΔCTR)::GFP]*
DNA76 *atgEx76[Punc25::hTauAP(ΔPRR)::GFP]*
DNA99 *atgEx99[Punc25::hTauCTR-AP3::GFP]*
DNA132 *atgEx132[Punc25::hTauAP::GFP + Punc25::EMTB::mKate2]*
DNA152 *atgEx152[Punc25::hTauS404A::GFP]*
DNA153 *atgEx153[Punc25::hTauS422A::GFP]*
DNA154 *atgEx154[Punc25::hTau::GFP+Punc25::EMTB::mKate2]*
DNA155 *atgEx155[Punc25::hTauS396A::GFP]*
DNA63 *atgEx163[TRE-Δpes-10-GFP::hTauAP+Punc25::rtetR-QFAD::P2A::mKate::T2A::tetR-pie1]*
DNA358 *atgEx358[Prgef-1::hTauAP(ΔMTB)::GFP]*
DNA359 *atgEx359[Prgef-1::hTauAP(ΔNTP)::GFP]*
DNA360 *atgEx360[Prgef-1::hTauAP(ΔPRR)::GFP]*
DNA363 *atgEx361[Prgef-1::hTauAP::GFP]*
DNA364 *atgEx364[Prgef-1::hTauE14::GFP]*
DNA365 *atgEx365[Prgef-1::hhTau::GFP]*
DNA366 *atgEx366[Prgef-1::hTauAP(ΔCTR)::GFP]*
DNA368 *atgEx368[Prgef-1::GFP]*
DNA61 *atgIs10[Psra-6::chrimson::sl2::mCherry]; atgEx2[Punc25::hTauAP::GFP]*
DNA62 *atgIs10[Psra-6::chrimson::sl2::mCherry]; atgEx3[Punc25::hTauE14::GFP]*

DNA63 *atgIs10[Psra-6::chrimson::sl2::mCherry]; atgEx4[Punc25::hTauWT::GFP]*
DNA64 *atgIs10[Psra-6::chrimson::sl2::mCherry]; atgEx5[Punc25::GFP]*
DNA65 *atgIs10[Psra-6::chrimson::sl2::mCherry]; atgEx72 [Punc25::hTauAP(ΔNTP)::GFP]*
DNA66 *atgIs10[Psra-6::chrimson::sl2::mCherry]; atgEx76[Punc25::hTauAP(ΔPRR)::GFP]*
DNA67 *atgIs10[Psra-6::chrimson::sl2::mCherry]; atgEx1[Punc25::hTauAP(ΔMTB)::GFP]*
DNA68 *atgIs10[Psra-6::chrimson::sl2::mCherry]; atgEx73[Punc25::hTauAP(ΔCTR)::GFP]*
DNA69 *atgIs10[Psra-6::chrimson::sl2::mCherry]; atgEx99[Punc25::hTauCTR-AP3::GFP]*
DNA610 *atgIs10[Psra-6::chrimson::sl2::mCherry]; atgEx155[Punc25::hTauS396A::GFP]*
DNA611 *atgIs10[Psra-6::chrimson::sl2::mCherry]; atgEx152[Punc25::hTauS404A::GFP]*
DNA612 *atgIs10[Psra-6::chrimson::sl2::mCherry]; atgEx153[Punc25::hTauS422A::GFP]*
